# The Asti Study: The Induction of Oxidative Stress in A Population of Children According to Their Body Composition and Passive Tobacco Smoking Exposure

**DOI:** 10.3390/ijerph16030490

**Published:** 2019-02-09

**Authors:** Giulia Squillacioti, Valeria Bellisario, Elena Grignani, Giulio Mengozzi, Giulia Bardaglio, Paola Dalmasso, Roberto Bono

**Affiliations:** 1 Department of Public Health and Pediatrics, University of Turin, 10126 Turin, Italy; giulia.squillacioti@unito.it (G.S.); valeria.bellisario@unito.it (V.B.); paola.dalmasso@unito.it (P.D.); 2 Maugeri Scientific Clinical Institutes, 27100 Pavia, Italy; elena.grignani@icsmaugeri.it; 3 City of Health and Science of Turin, Molinette Hospital, 10145 Turin, Italy; gmengozzi@cittadellasalute.to.it; 4 SUISM, Structure of Hygiene, Sport Sciences and Physical Activities, headquarters of Asti, University of Turin, 10126 Turin, Italy; giulia.bardaglio@gmail.com

**Keywords:** oxidative stress, children, Public Health, obesity, BMI, second-hand smoke

## Abstract

Obesity and exposure to second-hand tobacco smoking (SHS) may influence oxidative stress (OS) levels, especially in children. This study investigated body composition and SHS influence on OS induction in the paediatric population. The first purpose was identifying an appropriate BMI standard for adiposity assessment in OS investigations. Secondly, SHS and obesity were analysed as inductors of OS. The epidemiologic sample involved 330 children. Three BMI (body mass index) references (IOTF, CDC, and WHO) and an impedentiometric scale supplied body-composition measurements. Partecipants filled out a questionnaire and provided urinary samples for biomarker quantifications: isoprostane (15-F2t IsoP) and cotinine as OS and SHS biomarker, respectively. Obesity prevalence changed over different BMI references (14%, 21%, and 34% for IOTF, CDC, and WHO, respectively). Obese children, by IOTF, showed an increase of 56% in 15-F2t IsoP compared to those normal weight (*p* = 0.020). Children belonging to the third and the fourth cotinine quartile compared to those of the first quartile had higher 15-F2t IsoP (1.45 ng/mg, 95% CI: 1.06–1.97, *p* = 0.020 and 2.04 ng/mg, 95% CI: 1.55–2.69, *p* < 0.0001, respectively). Obesity assessment in children requires appropriate BMI reference depending on research field. Both SHS exposure and obesity may increase OS in children.

## 1. Introduction

Inflammation and oxidative stress (OS) are often considered as pre-pathological conditions originated from numerous non-optimal environments and working circumstances [1], from modifiable habits (e.g., tobacco smoking) [2,3] and from several health impairments which may represent both their cause and effect [4]. Various risk factors are able to induce redox imbalance in humans, and among these, adiposity [5,6] and second hand smoke (SHS) may play an important role in OS induction, in particular in youths [7,8,9]. Indeed, excessive adipose tissue and high exposure to SHS may induce a dysregulation of the balance between oxidants and antioxidants in favor of the oxidants [10,11], leading to OS [12], which determines a condition that often precedes the onset of several diseases [13,14]. Active tobacco smoking is able to promote oxidant production and to deplete the antioxidant defences of the body, and cigarette smoking has been related to a statistically significant decrease of the total antioxidant status, thus it may have a role in the mechanism by which tobacco smoking promotes inflammation [15]. SHS, to which more than 40% of children below 15 years of age are exposed [16], is associated with higher incidence of numerous health problems, including infections, asthma, and the decrease of lung function [3,17]. Moreover, free radicals may damage organs and tissues, playing a central role in passive-smoking-mediated disorders [9]. Environmental or household pollution can lead to increased severity and exacerbation of inflammatory diseases in children [18,19], who are the most vulnerable portion of the population as their respiratory, immune, and nervous systems are still in development [20]. Other factors, such as diet, qualitatively and quantitatively unsuitability, and scarce physical activity (PA), may adversely influence body composition, leading to overweight or obesity, which represent emerging issues worldwide, especially in the paediatric population [21,22]. The prevalence of overweight and obesity among children and adolescents aged 5–19 has risen from 4% in 1975 to over 18% in 2016 [23]. Childhood obesity is associated with higher future risks of obesity, premature death, and disability in adulthood. The excess of adipose tissue has been identified as a source of pro-inflammatory cytokines [24], which generate, in turn, further reactive oxygen species, “ROS”, in tissues, increasing the lipid peroxidation rate [25]. In obese subjects, adipose tissue determines an increase both in local and systemic production of pro-inflammatory adipocytokines that, in turn, induce OS [26]. Furthermore, OS promotes a systematic and systemic low-grade inflammatory response [27,28]. Inflammation of adipose tissue plays a critical role in the pathogenesis of obesity-related complications and several other diseases all associated with OS [28,29]. The Asti Study takes its name from the city of Piedmont, a region in the northwest part of Italy, where the subjects were recruited. It was designed to attain, in preventative terms, some of the essential objectives of Public Health in the paediatric population. In particular, this cross-sectional study aims to evaluate, in a large group of school-age children living in Asti, the role of body composition and SHS, as possible health risk factors able to influence the OS levels. The Asti study protocol provides details concerning the enrolment phases, the structuring of the questionnaire, the sampling of urine, and the biological and statistical analyses. OS has been evaluated by means of quantification of urinary isoprostane (15-F_2t_ IsoP), a prostaglandin-like compound derived from lipid peroxidation of arachidonic acid, widely used as an in vivo OS biomarker. Body composition was assessed by Body Mass Index (BMI) and Body Fat percentage (BF %). Moreover, to establish the most appropriate BMI standard in defining obesity condition of children in OS investigations, three different international references were selected and compared.

## 2. Materials and Methods

### 2.1. Study Area

Asti is a town of 76.211 inhabitants, 503 per km^2^, (01/01/2018 - ISTAT) located 123 meters above sea level in the Piedmont region, 55 km from Turin, the capital of Piedmont, the westernmost Region of the Po Valley. The Piedmont region, as well as the entire Padana Valley, is known for its high levels of atmospheric pollution due to the orographic position, the mountain range of the Alps, and the meteorological conditions, which led to the stagnation of the air masses reducing, at the same time, the dilution of pollutants. Based on these environmental characteristics, this specific study area deserves to be deeply investigated with regard to the relationship between airborne pollutants, OS, and respiratory health [30].

### 2.2. Ethics Committee Approval

All subjects gave their informed consent for inclusion before enrolment to the study. The study was conducted in accordance with the Declaration of Helsinki, and the Ethics Committee of Comitato Etico Internazionale A.O.U. Luigi Gonzaga (no. 0005540, tit. approved the protocol II, cat. 02, Cl. 01).

### 2.3. Selection of Subjects

The ASTI study is based on a population recruited from five primary schools located within the municipal boundaries of the city of Asti. All subjects participated as volunteers and they were enrolled according to the following inclusion criteria: only healthy children ranging from 8 to 11 years of age by March 2017, and resident in the selected area. Parents or legal tutors of each participant were asked to sign an informed consent and fill out a self-administered questionnaire. Then, each subject underwent the measurement of body composition and provided a urine sample for the quantification of: 15-F_2t_ IsoP, to quantify OS, and cotinine (COT) as biomarker of tobacco smoking exposure.

### 2.4. Questionnaire

The questionnaire was prepared according to the SIDRIA questionnaire [7,31] and was addressed and self-administered to the parents of the young volunteers. The questionnaire includes general information, address and residential area, house features, respiratory symptoms and allergies, family and socioeconomic status, SHS exposure, nutrition, physical activity, and physical inactivity.

### 2.5. Height

Height was estimated using a stadiometer (GIMA professional medical products) positioned on the wall at 2 meters of height in line with the surface base.

### 2.6. Impedance

Measurements of body composition were performed using an impedentiometric scale (FitScan BC-545F Tanita®), which adopts advanced Bioelectric Impedance Analysis (BIA) technology. Each subject was measured wearing light clothes and without shoes and socks. A skilled operator guided the subject onto the scale and recorded all data, expressed with decimal accuracy. For children (aged 7–17), the scale only displays three parameters, namely Body weight (kg), BMI (Kg/m^2^), and body fat (%) (BF). Two-compartment models, such as BIA, are capable to discern fat mass (FM) from fat free mass (FFM) in order to divide the total body mass into FM + FFM. We calculated FM index (FMI) expressed as kg/m^2^ from the ratio of BF to height squared and obtained FFM index (FFMI) as a complementary measure of FMI in BMI calculation.

### 2.7. BMI

To categorise subjects according to their body weight, BMI was used as measure of body composition. It was categorised according to three different classification systems proposed by the Centers for Disease Control and Prevention “CDC” [32], the International Obesity Task Force “IOTF” [33,34], and the World Health Organization “WHO” [35], respectively, and all cut-off values were sex and age-adjusted. Underweight (UW), normal weight (NW), overweight (OW), and obese (OB) categories were defined as (i) extrapolation of the adult BMI cut-off points according to the IOTF standard, (ii) based on centiles, in the case of the CDC reference, and (iii) according to standard deviations from the mean values of the WHO reference.

### 2.8. Urine

A spot of fresh urine was collected from each volunteer, aliquoted, and stored at −80 °C until analysis, performed within 2 months.

#### 2.8.1. Urinary 15-F_2t_ IsoP

The 15-F_2t_ IsoP was measured as biomarker of OS by E.L.I.S.A. technique according to manufacturer’s instructions (Oxford, MI, USA). The declared limit of detection is 0.2 ng/mL. Dilution 1:4 was made to achieve better assay accuracy. A preliminary incubation with β-glucuronidase for 2 h at 37 °C was performed to detect the entire quantity of 15-F_2t_ IsoP present in each urine sample, mostly excreted in human urine as glucuronic acid conjugated (over 50%) [36,37].

#### 2.8.2. Urinary Cotinine

COT was quantified as biomarker of tobacco smoking exposure as follows: transferring 10 mL of urine into a glass tube and adding 4.25 g of NaCl, 500 μL of NaOH (5 M), and 10 μL of cotinine-d3 as internal standard. Adding 2 mL of trichloromethane (CHCl_3_) to perform a double liquid–liquid extraction. Centrifuging the sample for 10 minutes at 3000 rpm and collecting the resulting organic phase a new glass tube; evaporating to dryness under a gentle steam of N_2_. The dry residue was reconstituted in 200 μL of CHCl_3_ and transferred into a conical vial for GC-MS determination. More details are described in previous studies [38].

#### 2.8.3. Urinary Creatinine

Creatinine was determined by the kinetic Jaffé procedure to normalise the excretion rate of all urinary biomarkers measured: 15-F_2t_ IsoP and COT.

### 2.9. Statistical Analyses

Descriptive analyses and comparison of socioeconomic characteristics, SHS, and body composition by gender and obese *versus* normal weight BMI classifications were carried out with the chi-square test for categorical variables (ethnicity and age classes), and the t test or Mann–Whitney U-test for quantitative parameters, as appropriate. The relationship between 15-F_2t_ IsoP, as a continuous dependent variable, and BMI classification according to IOTF, as an independent variable (NW as the reference category) was evaluated by Log-link Gaussian Generalised Linear Model (GLM), adjusted for cotinine quartiles, physical activity, gender, age, and body fat. Linear regression analyses were used to compare the relationship between BMI, as dependent variable and FMI, as independent variable, and the relationship between BMI as dependent variable and FFMI, as independent variable. All the tests were two-tailed and the level of significance was set at 0.05. All analyses were performed using STATA SE v14.2 (Stata Corp, College Station, TX, USA).

## 3. Results

The epidemiologic sample of this study consists of 330 children aged between 8 and 11 years. The description of the sample is provided in Table 1, which shows that the sample is homogeneous by gender, age, and ethnicity. Males and females were also compared with each other for height, weight, and body composition (described by BMI as continue variables, BF %, and FMI-FFMI body components).

**Figure 1:**
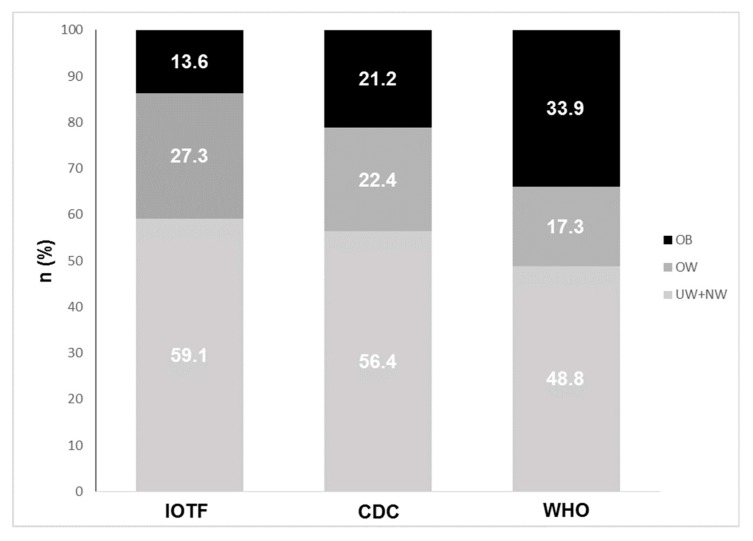
Prevalence of normal weight “NW”, over weight “OW”, and obese “OB” children categorised by three different BMI references: IOTF, CDC, and WHO.

Only BF % is significantly higher (*p* < 0.0001) in females. Figure 1 shows children categorisations based on three selected international BMI standards and referred to their principal sub-groups: NW, OW, and OB. Underweight group was less than 5% of the whole sample in each BMI standard, thus it has been considered together with the NW group. The prevalence of obesity varies using different BMI standards—WHO reports the highest numerousness of obese children, CDC categorises almost equally OW and OB, and IOTF shows the lowest prevalence of obesity. Moreover, 15-F_2t_ IsoP distribution does not change over UW+NW, OW, and OB categories, whereas there is a tendency of 15-F_2t_ IsoP increase from UW+NW to OB category. Levels of 15-F_2t_ IsoP between IOTF standard categories are equal to 4.5 ± 3.8, 4.9 ± 3.9, and 5.7 ± 4.7 ng/mg Crea in UW+NW, OW, and OB, respectively (*p* = 0.091). According to CDC categorisations, OS biomarker levels are 4.3 ± 3.8, 4.8 ± 3.4, and 5.3 ± 4.7 ng/mg Crea in UW+NW, OW, and OB children (*p* = 0.356). The WHO categories of UW+NW, OW, and OB correspond to 4.4 ± 3.9, 5.0 ± 3.3, and 5.1 ± 4.3 ng/mg Crea of 15-F_2t_ IsoP, respectively (*p* = 0.216). Although no difference in 15-F_2t_ IsoP distribution is significant among UW+NW, OW, OB groups, the IOTF standard shows the nearest significance level, reporting a *p*-value of 0.091.

Table 2 reports the results of the Generalised Linear Model (GLM), adjusted by SHS as confounder, sex, age, BF %, and PA. Both being obese by IOTF standard and exposed to SHS determines an increase in urinary 15-F_2t_ IsoP.

**Figure 2:**
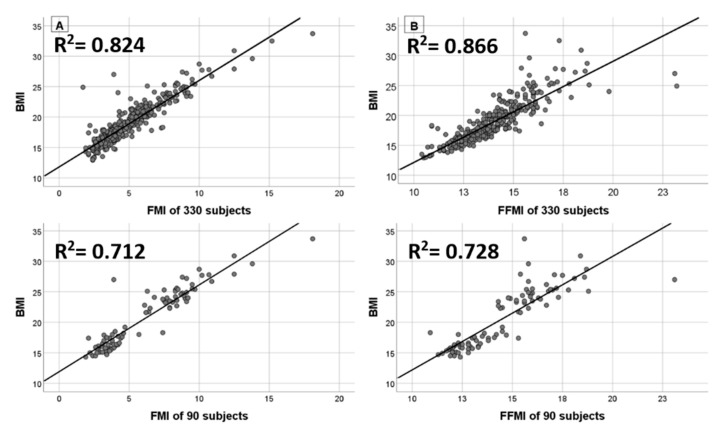
Linear regression between BMI and FMI on the left (**A**), and BMI and FFMI on the right (**B**). The upper-side scatterplots refer to the whole sample (n = 330) and the lower-side scatterplots refer to the subgroup of children categorised in accordance with all BMI standards simultaneously (n = 90).

In order to establish whether BMI was a reliable proxy of obesity status in our sample, we also analysed BMI correlations and increments with respect to contributions of the two body-composition compartments: FMI and FFMI. As shown in Figure 2, BMI is significantly and positively correlated to both FMI and FFMI (*p* < 0.0001). Furthermore, in order to calculate BMI gradients, a subgroup of children (*n* = 90), who have been categorised OB and NW by all the three standards simultaneously, were selected from the whole sample. A number of 45 obese subjects was considered as reference to randomly select a homogeneous sample, in terms of sex and age, of 45 other children categorised as NW by all the three standards.

Table 3 shows as FMI and FFMI mean values are significantly higher in OB children than in those who are NW (*p* < 0.0001). Moreover, 15-F_2t_ IsoP displays significant differences between two groups, with higher levels in OB subjects, for both Ln-transformed values and not-transformed, *p* = 0.002 and *p* = 0.038, respectively. Taking into account FMI and FFMI average differences in OB and NW children, we calculated gradients to assess whether BMI higher levels were mostly due to an average increase of FMI, thus adiposity, or on the contrary, a greater average raise in the amount of FFMI and to support results displayed in Figure 2. FMI and FFMI mean values of the gradients are statistically different and in particular FMI has the highest gradient mean value equal to 5.2 ± 2.5 Kg/m_2_ compared to FFMI gradient mean value of 3.5 ± 1.9 Kg/ m^2^ (*p* < 0.0001).

## 4. Discussion

Obesity is a health worldwide issue, affecting both adults and children. This multi-factorial condition represents a risk factor for many diseases and predisposes children to chronic adulthood health problems, such as metabolic syndrome, diabetes mellitus, cardiovascular diseases, and cancer [23]. Adipose tissue has been identified as an active endocrine organ involved in the production of adipocytokines or adipokines [39]. Low-grade inflammation and OS are two of the key mechanisms implied in the obesity-induced metabolic complications [5] and in worsening obesity condition itself [28]. Moreover, excessive OS may damage cellular structures and influence the anti-oxidant defences which can be compromised and do not counteract redox disequilibrium [12].

The purpose of this study was to investigate whether body composition, and in particular, obesity condition, assessed by BMI, may influence urinary 15-F_2t_ IsoP levels and OS status in healthy children. Moreover, in order to characterise obese subjects, three international BMI references were selected, since the percentage of youths classified as overweight or obese varies considerably, depending on the BMI cut-points or centiles [40]. The three BMI standards highlighted significant differences in categorising children between categories. Indeed, the application of the IOTF standard has highlighted the smallest percentage of obese children compared to the CDC standard (14% vs 21%) and even wider difference compared to the WHO standard (14% vs 34%). Our results are in keeping with those reported by many other studies [41,42,43].

With regard to NW and OB children, categorised by all three standards simultaneously, overall 14% (*n* = 45) of the sample has been classified as obese and 44% (*n* = 144) were considered NW, but for the calculation of the BMI gradients, only 90 subjects (45 OB + 45 NW) were considered. By analysing the role of obesity in OS induction, this study highlights a higher effectiveness of the IOTF standard in categorising children as obese with the final purpose of investigating OS intensity. Whereas isolated analyses on BMI categorisations and OS induction do not show significant differences between groups, the fully adjusted multivariate model pinpoints that obese children, by the IOTF reference, have higher levels of urinary 15-F_2t_ IsoP. Being obese promotes an increase of 56% in 15-F_2t_ IsoP levels compared to those measured in normal weight children (*p* = 0.020). Interestingly, statistical analyses that took into account the other two standards did not show any results about the relationship between obesity and OS. Only IOTF seems to clarify the role of adiposity in OS induction, showing an appropriate accuracy. This result is independent from another important OS predictor: SHS exposure, which has been considered as a confounder in the GLM model. Nonetheless, SHS shows its influence on OS and children who belong to the third and the fourth COT quartile compared to those of the first quartile, who have higher 15-F_2t_ IsoP levels (1.45 ng/mg, 95% CI: 1.06–1.97, *p* = 0.020 and 2.04 ng/mg, 95% CI: 1.55–2.69, *p* < 0.0001, respectively).

In general, by considering SHS exposure as the independent variable in OS induction, children exposed to the highest levels of SHS exhibit higher levels of urinary 15-F_2t_ IsoP (*p* < 0.0001) and subgrouping COT in quartiles, the fourth quartile corresponds to the highest level of 15-F_2t_ IsoP. Concerning body-composition analyses, it can be noticed that BMI is positively and significantly related to both FMI and FFMI components. Interestingly, linear regression between BMI and FMI accounts for a greater value of explained variance equal to 0.824 (*n* = 330) and 0.866 (*n* = 90), which are higher than those related to BMI and FFMI regression (0.712 and 0.728 for *n* = 330 and *n*= 90, respectively). This result highlights a better fit between BMI and FMI, which in turn, reflects adiposity, and supports the usage of BMI as a proxy of obesity condition in this sample. This sub-classification also allows understanding whether BMI may be considered as a proxy of adiposity in the current study and supports its usage in body composition inquires. In this context, investigations on the relationship between FMI, FFMI, and increased BMI values highlights that higher BMIs are more influenced by an average increment of FMI than FFMI, thus the FMI component in BMI of obese children is, on average, greater than FFMI. Further analyses on the subgroup of children categorised in accordance to all three BMI standards show that OS biomarker levels are significantly different between OB children compared to those of NW. In particular, being obese, per se, implies increased levels of urinary 15-F_2t_ IsoP (*p* = 0.002).

In summary, both SHS and body composition, particularly obese condition, are influencing factors in OS induction. Hence, we observed that school-aged children showed higher levels of urinary 15-F_2t_ IsoP in relation to both adiposity and SHS exposure, which are independent OS predictors. In the fully adjusted multivariate model, BF %, considered as a continuous variable, did not display any influence on OS biomarker. The difference of OS observed between genders, where females show higher BF % than males (*p* < 0.0001), may be due to those girls who underwent early pubertal maturation, but no differences were observed in OS biomarkers between males and females. Future investigations could be focused on body fat profile changes over time, e.g., in a longitudinal study, where BF is considered to be more appropriate than BMI [44]. Nevertheless, our study aimed primarily to analyse BMI because of its easy measurement, cost-effectiveness, and high correlation with other tools for body composition assessment in epidemiologic studies [45]. It was valuable in establishing which BMI reference was more appropriate for OS investigations and obesity in children. Whereas all three BMI standards are effective tools in prevention strategies, their differences in accuracy, sensitivity, and specificity requires specific fields of application and IOTF appears more appropriate in OS inquiries.

Although specific fields of application deserve the most appropriate BMI reference, prevention strategies in children for public health are adequately supplied by all these BMI standards, which are valuable tools in primary prevention against obesity. Hence, all three references show their utility in defining obese children, even though they may show different sensitivity and specificity depending on the field of application. The main limitation of this study is its cross sectional design that did not allow us to explore OS and body composition changes over time and their causal relationship. Thus, only differences in obesity prevalence could be described and associated with OS induction. Furthermore, BF has been measured by the BIA method, whose prediction equation assumes 73% of water as hydration of the participants [46], which does not always reflects real sampling conditions and this aspect cannot be overcome. On the other hand, the BIA has been considered as the simplest and least expensive method for BF evaluation in clinical practice [46].

## 5. Conclusions

In conclusion, urinary concentration of 15-F_2t_ IsoP is significantly higher in children classified as obese by all three BMI references together as well as by IOTF standard, compared to those who were categorised as normal weight. Thus, OS is influenced by body composition, in particular by adiposity. BMI represents a suitable tool in epidemiologic study investigations on OS and a valuable instrument in screening the school-aged populations to support and promote public health, with the final purpose of defining the best prevention strategies against obesity and risky conditions for health.

## Figures and Tables

**Table 1:** Descriptive and physical characteristics of the epidemiologic sample.

		Females *n* = 161 (48.8%)	Males *n* = 169 (51.2%)	*p*-Value	Total 330
Age (years)	8	51 (31.7)	56 (33.1)	0.84	107 (32.4)
	9	58 (36.0)	53 (31.4)		111 (33.6)
	10+	52 (32.3)	60 (35.5)		112 (33.9)
Ethnicity (n)	Non-Caucasian mothers ^a^	15 (9.3)	17 (10.6)	0.32	32 (9.7)
	Non-Caucasian fathers ^a^	17 (10.1)	17 (10.1)	0.54	34 (10.3)
Height (cm)		138.4 ± 9.3	138.9 ± 8.4	0.57	138.4 ± 8.7
Weight (kg)		36.5 ± 10.1	36.8 ± 10.8	0.78	36.3 ± 10.2
BMI (kg/m^2^)		19.1 ± 3.6	18.8 ± 3.6	0.28	18.8 ± 0.2
FMI (kg/m^2^)		5.2 ± 2.2	4.8 ± 2.3	0.07	5.0 ± 2.3
FFMI (kg/m^2^)		13.9 ± 1.9	14.0 ± 1.6	0.44	14.0 ± 1.8
Body Fat (%)		26.9 ± 6.2	24.3 ± 6.6	<0.0001	25.4 ± 6.5

Notes: Height, weight BMI, Body Fat %, FMI and FFMI are expressed as mean ± SD. ^a^ Non-Caucasian includes African, Asiatic, and Hispanic ethnicities.

**Table 2:** Generalised linear model with 15-F_2t_ IsoP as dependent variable, fully adjusted for sex, age, body fat percentage, physical activity, BMI categories by IOTF, and cotinine quartiles.

15-F_2t_ IsoP	Exp (β) (95% C.I.)	*p*-Value
Body composition ^a^:		
Overweight (IOTF)	1.22 (0.97–1.56)	0.095
Obese (IOTF)	1.56 (1.07–2.27)	0.020
Cotinine quartiles ^b^:		
COT 2^nd^ quartile	1.27 (0.93–1.72)	0.130
COT 3^rd^ quartile	1.45 (1.06–1.97)	0.020
COT 4^th^ quartile	2.04 (1.55–2.69)	<0.0001
Physical activity ^c^:		
Moderate	1.00 (0.83–1.23)	0.944
Intense	1.14 (0.81–(1.61)	0.440
General characteristics ^d^:		
Sex	1.09 (0.92–1.31)	0.297
Age	1.06 (0.96–1.15)	0.210
Body fat (%)	1.00 (0.97–1.01)	0.110

^a^ Body composition expressed through BMI categories by IOTF cut-offs (UW+NW as reference group. ^b^ Cotinine: 1^st^ quartile as reference. ^c^ Physical activity: up to one day/week as reference, “moderate” = 2–4 days/week and “intense” = 5–7 days/week. ^d^ sex: female as reference gender; BF%: as continue variable.

**Table 3:** Subgroup of children categorised as NW and OB by all standards simultaneously.

	Normal Weight *n* = 45	Obese *n* = 45	*p*-Value	Gradients ∆
AGE (years)	9.2 ± 0.9	9.2 ± 1.0	0.81	
FMI (Kg/m^2^)	3.6 ± 0.9	8.8 ± 2.3	<0.0001	5.2 ± 2.5
FFMI (Kg/m^2^)	12.8 ± 0.9	16.3 ± 1.6	<0.0001	3.5 ± 1.9
15-F_2t_ IsoP (ng/mg Crea)	3.8 ± 3.7	5.7 ± 4.7	0.039	4.1 ± 4.8
Ln(15-F_2t_ IsoP) (ng/mg Crea)	0.99 ± 0.79	1.50 ± 0.67	0.002	0.8 ± 0.6

Values are expressed as mean ± SD.
